# Maternal Docosahexaenoic Acid Intake Levels During Pregnancy and Infant Performance on a Novel Object Search Task at 22 Months

**DOI:** 10.1111/cdev.12280

**Published:** 2014-08-11

**Authors:** Alison Rees, Sylvain Sirois, Alison Wearden

**Affiliations:** 1 The University of Manchester; 2 Université du Québec à Trois-Rivières

## Abstract

This study investigated maternal prenatal docosahexaenoic acid (DHA) intake and infant cognitive development at 22 months. Estimates for second- and third-trimester maternal DHA intake levels were obtained using a comprehensive Food Frequency Questionnaire. Infants (*n* = 67) were assessed at 22 months on a novel object search task. Mothers' DHA intake levels were divided into high or low groups, with analyses revealing a significant positive effect of third-trimester DHA on object search task performance. The third trimester appears to be a critical time for ensuring adequate maternal DHA levels to facilitate optimum cognitive development in late infancy.

This study examines maternal docosahexaenoic acid (DHA) intake levels during pregnancy in relation to infant postnatal cognitive development. DHA is a long-chain member of the omega-3 (n-3) family of polyunsaturated fatty acids (PUFAs). DHA is available in the diet primarily in oily fish such as mackerel and also in supplement form. DHA and other long-chain n-3 PUFAs can also be synthesized (albeit, highly inefficiently; see Plourde & Cunnane, 2007) in the body from the short-chain parent compound of the n-3 family, alpha linolenic acid.

## DHA During Pregnancy

Long-chain polyunsaturated fatty acids (LCPUFAs) are highly concentrated in the myelin sheath and membranes of synaptic terminals of the brain and central nervous system (see Sastry, 1985; Yehuda, Rabinovitz, & Mostofsky, 2005). During the third trimester of pregnancy, a period of dynamic structural neural growth occurs in the fetal brain, coinciding with a huge increase in the selective accumulation of DHA, which is preferentially transferred across the placenta (Hanebutt, Demmelmair, Schiessl, Larqué, & Koletzko, 2008), indicating a particular need for this nutrient at this time (Hornstra, 2000; Innis, 2000, 2007a, 2007b). Prenatally, maternal diet during pregnancy is a key in determining levels of DHA available to the fetus.

### DHA and Cognitive Development

Higher levels of LCPUFAs are thought to have particular benefits in the development of cognitive functions during infancy. Several studies report positive effects of higher DHA (either postnatally in the infant or prenatally in the mother) and improved performance on a range of cognitive outcomes such as problem solving (e.g., Willatts, Forsyth, DiModugno, Varma, & Colvin, 1998), attention (e.g., Colombo et al., 2004; Colombo et al., 2011; Kannass, Colombo, & Carlson, 2009), habituation (e.g., Colombo et al., 2004), and general tests of cognitive development (e.g., Helland, Smith, Saarem, Saugstad, & Drevon, 2003; Jacobson et al., 2008). However, other studies report no benefit of higher DHA (e.g., Auestad et al., 2003) or negative effects relating, specifically, to language (e.g., Drover et al., 2012).

Variations in study design are thought to contribute to the variability in findings with several researchers in the field stressing the importance of focusing on specific cognitive systems rather than global measures (e.g., Colombo, 2001) in order to properly elucidate effects of DHA on cognitive functioning (e.g., Cheatham, Colombo, & Carlson, 2006).

### Objectives and Rationale for the Current Study

The objective of this study was to examine prenatal maternal LCPUFA levels, particularly DHA, in relation to infant cognitive development at 22 months. The study is unique in its attempt to determine whether there is a critical time point during pregnancy at which DHA intake levels affect cognitive development by comparing effects of second- and third-trimester levels. Fetal neural development is very different at each of these stages; therefore, varying DHA intake levels may affect cognitive development differentially at these points. To aid development of practical nutrient guidelines for pregnant women, the study employed a Food Frequency Questionnaire (FFQ) to determine maternal LCPUFA levels rather than physiological measures, as any beneficial effects on cognitive development could be directly linked to dietary intake and not just physiological measures of a nutrient, which may have numerous genetic and metabolic determinants (e.g., Anil, 2007; Brenna, Salem, Sinclair, & Cunnane, 2009).

The infant assessment session took place at 22 months postpartum. Testing later in infancy (usually defined as up to 2 years old) allows for more complex tasks to be used due to more advanced understanding, higher cooperation, and a greater attentional window. A novel object search task was developed following a comprehensive review of prior work with both human infants and comparative studies. Object search taps a number of cognitive skills including working memory, short-term memory, visuospatial skills, and attention, and requires the infant to have a developed sense of object permanence and partake in goal-directed behavior. It is therefore a suitable task for the assessment of overall infant cognitive development at this age.

We predict that higher maternal DHA intake levels during pregnancy will result in better performance on an object search task at 22 months. Moreover, if there is a critical prenatal time point at which DHA intake levels result in significantly better performance, this will be the third trimester when DHA accretion is rapid.

The data reported here form part of a larger longitudinal study, for which a number of additional measures were taken (including further data collection points at 4.5 and 9 months postpartum). However, these are not part of this specific subproject and are not considered further in this report.

## Method

### Participants

A total of 125 women, both primiparous and multiparous, experiencing a single healthy pregnancy were recruited from a catchment area within a 30-mile radius from the University of Manchester. Recruitment strategies included leafleting at numerous venues, media coverage, features and adverts in local magazines and newspapers, recruitment e-mails aimed at university staff members and students, and a Web site dedicated to the study.

The study was approved by the University of Manchester's School of Psychological Sciences Research Ethics Committee and multiple consent forms were signed by the mother at several different stages of the study.

### Design

**Figure 1:**
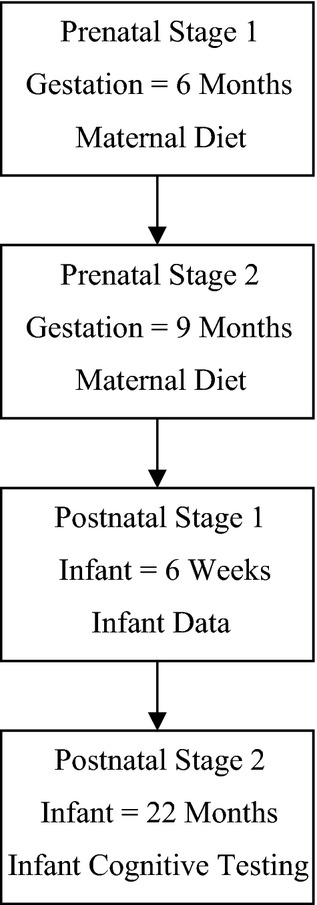
Illustration of the data collection points of the study.

A longitudinal study design was employed with data collection points as shown in Figure1.

### Procedure, Materials, and Apparatus

#### Prenatal Stage 1—Gestation = 6 Months

Women completed the first stage of the study between their 21st and 28th week of pregnancy following a 20-week scan, which detects the majority of fetal abnormalities. Ninety-three (74.4%) women were assessed at home and 32 (25.6%) at the University of Manchester Babylab. Mean gestation at assessment was 24.56 weeks (range = 21–28 weeks, *SD* = 2.10).

##### Maternal questionnaire measures

Maternal dietary information was obtained from the Diet History Questionnaire (DHQ, developed by the National Cancer Institute [NCI], U.S.), shown in a number of validation studies (e.g., Subar et al., 2001) to provide reasonable nutrient estimates for a range of n-3 and n-6 PUFAs. The DHQ has 149 questions that focus on frequency of consumption and approximate portion sizes of various foods, drinks, and dietary supplements. The DHQ was adapted to allow examination of a 3-month (i.e., single trimester) period and for a U.K. participant group. Alongside the DHQ, five supplementary dietary questions that specifically asked about oily fish, and also included information on serving size, were asked. These were taken from an FFQ that has been validated (via erythrocyte concentrations) as providing accurate estimates of n-3 LCPUFAs in early pregnancy (Fawzi, Rifas-Shiman, Rich-Edwards, Willett, & Gilman, 2004) and has been used in an earlier study assessing fish intake in pregnancy in relation to infant cognitive development (Oken et al., 2005).

#### Prenatal Stage 2—Gestation = 9 Months (35–37 Weeks)

The procedure at this visit was identical to the 21- to 28-week gestation stage; 99 (79.2%) mothers were assessed at home and 16 (20.8%) at the Babylab. Mean gestation was 36.15 weeks (range = 35–37 weeks, *SD* = 0.84).

##### Maternal questionnaire measures

The DHQ and supplementary fish intake questions were again completed. An additional questionnaire was administered asking about PUFA supplements that may have been taken before and during pregnancy; this included frequency, duration, manner of supplementation, and detailed product information.

Participants were given two forms to be returned to the researcher following the birth of their baby: a second consent form and a questionnaire about their newborn. If these forms had not been received 3 months past each participants' expected due date then a reminder letter was issued.

#### Postnatal Stage 1—Initial Infant Data Collection = 6 Weeks

Within 6 weeks, postpartum mothers returned the questionnaire that provided initial data about their newborn. This asked about infant birth weight, head circumference, length, sex, birth date, health, and about the labor and birth itself.

#### Postnatal Stage 2—Infant Cognitive Assessment = 22 Months

All infant assessment took place at the Babylab at the University of Manchester at times when infants were judged to be alert and content (i.e., not hungry, tired, or unwell). The mean age of infants at testing was 665 days (21.6 months), range = 610–724 days (19.8–23.5 months), *SD* = 28.4.

##### Object search task

We developed this task based on similar, age-appropriate tasks in the literature (e.g., Perlmutter et al., 1981; Wellman, Ritter, & Flavell, 1975).

**Figure 2:**
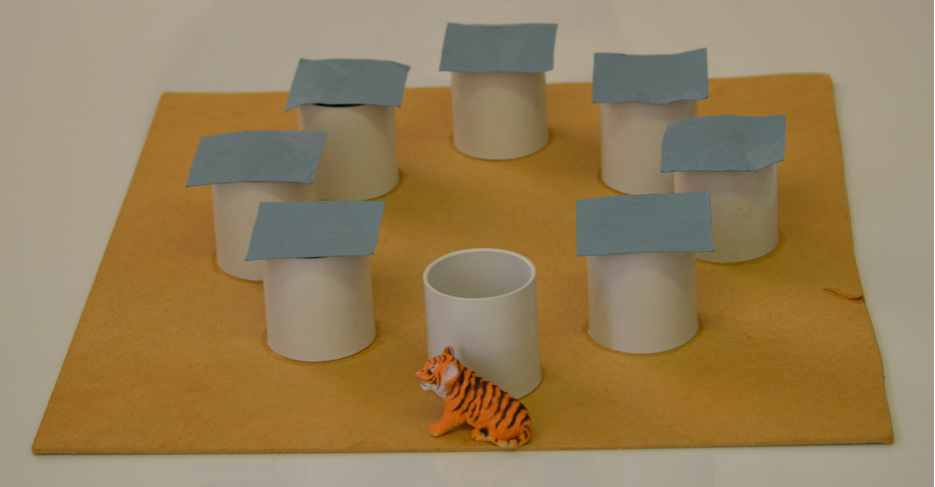
Apparatus used for the object search task.

Infants sat on their mothers' knee at a table with the researcher sat opposite. The apparatus was placed on the table in front of the infant (see Figure2). This consisted of a piece of wooden board roughly 0.5 m^2^ on which eight open-top cylinders (diameter 6 cm) had been glued equidistant in a circular pattern. Each cylinder had a square piece of cardboard placed on top of it to fully conceal the open top. A small toy tiger was the object to be hidden and a Thomas the Tank Engine toy was used for distraction.

Eight trials took place (one for each cylinder) and the order in which the tiger was hidden was identical for every infant. For each trial the researcher showed the infant the tiger and said, “[Child's name] look at this tiger—he's a lovely tiger but he's feeling very tired. Why don't we let him have a little sleep in here?” When the infant's gaze was directed on the tiger the researcher placed it in the appropriate cylinder and covered the cylinder with the cardboard. While doing this the researcher said to the infant “Look, in he goes, let's cover him up, night-night Mr. Tiger, night-night.” The infant was then distracted for 30 s using the Thomas the Tank Engine toy, which could be pulled back and released to travel across the table to the infant. The infant was asked to catch Thomas and then send the train back across the table to the researcher. After 30 s the researcher said, “[Child's name], where's that little tiger who went for a sleep? Can you find him for me?” If the child failed to indicate a location selection after 20 s had passed the researcher repeated the question.

Mothers were asked not to help their infant retrieve the tiger, but they could assist in the distraction part of the task if necessary. The infant was scored by the experimenter as the task took place and scoring was based on the cylinder that they selected first. A correct selection gained maximum points (4) and the score was reduced by 1 point for each cylinder further away from the correct one. Infants were encouraged to complete all eight trials, but if they were fussy or lost interest, the maximum number possible was completed. As a number of infants refused to complete all eight trials, a total score based on the first four trials was included in the analysis so as to include an outcome measure for all infants. Total overall score, mean scores, and number of trials completed were also calculated for analysis.

##### Maternal questionnaire measures

The Home Screening Questionnaire (HSQ; Frankenburg & Coons, 1986) containing a number of questions relating to the home environment was administered to mothers. The HSQ has been successfully used in a number of similar studies (e.g., Zhou, Baghurst, Gibson, & Makrides, 2007). It comes with scoring criteria and produces an overall score from 0 to 43.

Mothers also completed a breastfeeding questionnaire, which included a question on duration of breastfeeding.

### Data Processing

As a number of the participants were taking fish oil supplements containing DHA and EPA, the values used for these nutrients in all analyses consisted of the *total* DHA and EPA amounts, that is, the sum of the supplemental intake (calculated per day, for each participant, using values from the specific brand used and the number of times taken per day per week) and the DHQ estimate for dietary daily values.

The dietary data files collected from the DHQ were converted to nutrient files following the prescribed procedure using the Diet*Calc software package developed by the NCI (Bethesda, MD) and provided to users of the DHQ for this purpose. This procedure generated a file that contains daily nutrient (including DHA) and food group estimates for each participant at each point of testing.

For each nutrient to be used in the analysis, participants' individual values were recoded into two groups—low and high—using a median split; this was performed separately for second- and third-trimester data. The median DHA intakes, in grams per day, were 0.08 and 0.09 for the second and third trimesters, respectively. Values ranged from 0.0 to 0.46 and 0.0 to 0.56 for the same trimesters. Mean second-trimester DHA intakes for the low group were 0.067 (*SD* = 0.078) and 0.076 (*SD* = 0.085) for the second and third trimesters, respectively. For the high groups, the corresponding values were 0.195 (*SD* = 0.122) and 0.186 (*SD* = 0.143). Mean third-trimester DHA intakes for the low group were 0.080 (*SD* = 0.091) and 0.067 (*SD* = 0.093) for the second and third trimesters, respectively. For the high groups, the corresponding values were 0.167 (*SD* = 0.127) and 0.177 (*SD* = 0.131). Comparisons using one-way analyses of variance (ANOVAs) were carried out to check for homogeneity of the high- and low-DHA groups across a number of parental and infant characteristics for both second- and third-trimester data. Parental variables examined as potential covariates were maternal age, maternal and paternal years in education (number of years in education since beginning secondary school at age 11), and prepregnancy body mass index (kg m^−2^). Infant variables were birth weight, length, and head circumference; gestation length; and Apgar scores at 1 and 5 min following delivery. Mean duration of breastfeeding, infant age at testing, and HSQ scores were also examined as potentially confounding factors.

## Results

### Retention Rate

Some participants were dropped out of the study at each time point. Table1 shows the retention rate at each stage. Reasons for leaving the study were as follows: nonreturn of postbirth forms (*n* = 14), repeatedly unable to be contacted (*n* = 7), relocation (*n* = 9), premature birth (*n* = 2), and changed mind about participation (*n* = 1). The majority of participants (*n* = 25) were lost due to the researcher being on maternity leave and unable to test infants when they reached the appropriate age.

**Table 1:** Number and Percentage of Participants Remaining in the Study at Each Point of Contact

	*n*	%
Retained at second pregnancy visit	115	92.0
Retained at 6 weeks postpartum	107	85.6
Retained at 22 month infant visit	67	53.6

To check for differences between the retained sample and those participants who dropped out, comparisons of group means were performed using one-way ANOVAs for a number of variables. The outcomes are shown in Table2. The two groups differ with respect to infant head circumference (with the retained group having a significantly smaller head circumference at birth compared to the dropout group) and Apgar score at 5 min postbirth (with retained group scoring significantly higher).

**Table 2:** Comparison of Variable Measures Between the Dropout Group and Retained Group of Participants at 22 Months

	Dropout	Retained	*p*
Parental characteristics			
Maternal age (years)	33.7 (4.1)	32.8 (4.0)	.24
Paternal age (years)	35.4 (5.5)	35.1 (4.4)	.72
Prepregnancy body mass index	24.2 (3.8)	23.7 (4.1)	.51
Maternal education (years)	10.2 (2.4)	10.7 (2.4)	.20
Paternal education (years)	9.19 (2.8)	9.84 (2.6)	.19
Infant characteristics			
Gestation length (days)	283.7 (9.4)	282.0 (8.5)	.32
Weight (kg)	3.7 (0.4)	3.6 (0.5)	.12
Head circumference (cm)	35.4 (1.2)	34.8 (1.3)	.03*
Length (cm)	54.5 (2.9)	53.1 (3.4)	.09
Apgar score (1 min)	8.3 (1.7)	8.7 (1.0)	.14
Apgar score (5 min)	8.9 (1.6)	9.5 (0.8)	.04*

Note

The mean value for each group (standard deviation in parentheses) is given along with the *p* value for comparison of group means.

* *p* < .05.

### Pregnancy and Birth Data

One hundred and seven questionnaires were returned, reporting 55 (51.4%) female infants born and 52 (48.6%) male. Gestation length (days) was calculated from the difference between the birth date and due date, with a standard pregnancy taken as lasting 280 days. Mean gestation length was 282.63 days (range = 238–296 days, *SD* = 8.85). Of 107 births, 86 (80.4%) were via vaginal delivery, 10 (9.3%) via elective Cesarean, and 11 (10.3%) via emergency Cesarean.

### Preliminary Analyses

Table3 shows the high- and low-DHA groups used in the main analyses. The two groups did not differ on any variable other than DHA intake levels.

**Table 3:** Mean Values (Standard Deviation) of Parental and Infant Characteristics for the High- and Low-Docosahexaenoic Acid Groups Used in the Analyses

	Second trimester			Third trimester		
	Low	High	*p*	Low	High	*p*
Parent characteristics						
Maternal age (years)	32.6 (3.9)	33.9 (4.3)	.09	32.8 (4.1)	33.6 (4.1)	.28
Prepregnancy body mass index (kg m^−^²)	24.2 (4.6)	23.7 (3.5)	.53	23.8 (3.8)	24.2 (4.5)	.58
Maternal education (years)	10.1 (2.5)	10.9 (2.2)	.08	10.1 (2.6)	10.9 (2.2)	.08
Paternal education (years)	9.4 (2.6)	9.6 (2.6)	.75	9.8 (2.7)	9.6 (2.6)	.76
Infant characteristics						
Weight (kg)	3.7 (0.4)	3.6 (0.5)	.38	3.7 (0.5)	3.6 (0.4)	.69
Head circumference (cm)	35.0 (1.3)	34.9 (1.4)	.82	34.9 (1.1)	35.1 (1.4)	.49
Length (cm)	53.8 (3.1)	53.0 (3.2)	.30	53.4 (3.3)	53.7 (3.4)	.77
Apgar score (1 min)	8.7 (0.7)	8.5 (1.4)	.49	8.6 (0.6)	8.5 (1.4)	.76
Apgar score (5 min)	9.3 (0.8)	9.2 (0.8)	.61	9.3 (1.0)	9.3 (0.5)	.92
Gestation length (days)	282.7 (9.2)	280.8 (7.6)	.91	282 (9.4)	284 (7.5)	.21
Duration breastfed (months)	9.3 (3.9)	9.2 (3.7)	.96	8.9 (3.8)	9.5 (3.6)	.73
Age at testing (months)	667.0 (4.7)	662.5 (5.0)	.49	660.1 (4.6)	670.0 (4.9)	.17
Other factors						
Home screening Questionnaire score	36.5 (2.2)	37.3 (2.0)	.15	36.5 (2.5)	36.9 (2.0)	.48

Note

Comparison of group means was carried out with one-way analyses of variance and the *p* values are given below.

### Main Analyses

One-way ANOVAs were performed with DHA group as the independent variable and task outcome measures as dependent variables. The results of the ANOVAs are shown in Table4.

**Table 4:** Mean (Standard Deviation) and *p* Values for Each of the Infant Cognitive Outcome Measures, for the Second and Third Trimester Low- and High-Docosahexaenoic Acid Groups

	Second trimester			Third trimester		
	Low	High	*p*	Low	High	*p*
Object search task						
Trials completed	7.3 (1.4)	7.4 (1.3)	.74	7.3 (1.4)	7.5 (1.2)	.56
Total score (first four trials)	12.8 (2.0)	13.0 (2.4)	.78	12.3 (2.1)	13.6 (2.1)	.03*
Total score (all trials)	22.0 (5.9)	22.9 (5.2)	.53	21.3 (5.9)	23.9 (4.8)	.07
Mean score (over all trials)	3.0 (0.6)	3.1 (0.4)	.51	2.9 (0.5)	3.2 (0.4)	.05*

* *p* < .05.

The analysis revealed a significant effect of third trimester DHA with mean score, *F*(1, 61) = 3.87, *p* = .05, 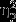 = 0.06, observed power = 0.49, and total score for the first four trials, *F*(1, 61) = 5.25, *p* = .03, 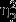 = 0.08, observed power = 0.62. Total score over all eight trials almost reached significance, *F*(1, 61) = 3.53, *p* = .07, 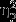 = 0.06, observed power= 0.46. In all cases homogeneity of variances was assumed. Examination of the group means revealed the high-DHA group to be performing best in all three of these measures.

The same analyses were also carried out with EPA and AA. There were no significant findings for either nutrient, at either trimester, on any of the cognitive outcome measures.

## Discussion

We found that infants whose mothers were in the high-prenatal maternal DHA group had better performance on an object search task at 22 months than those with mothers in the low-prenatal DHA group, adding further support to prior findings (e.g., Colombo et al., 2004; Helland et al., 2003). Furthermore, this effect was only significant when related to third-trimester DHA intake levels, thus uniquely demonstrating that the critical time point for maternal DHA intake levels in relation to infant cognitive development is during the third trimester. The third trimester marks the beginning of a substantial rise in the uptake of DHA in fetal brain, which continues to increase rapidly until birth. Higher maternal intake then possibly results in the accumulation of greater levels of DHA in the membranes of synaptic terminals or increased myelination, which may facilitate faster information processing during cognitive tasks.

The object search task was specially designed for this study, so it is not possible to say how the between-group difference we report compares with other findings in the literature. However, it appears that our task is an appropriate cognitive assessment for use in studies examining differences in DHA levels in relation to cognitive development later in infancy. The task also has the benefit of being short and easy to administer, requiring little specialist equipment.

### Study Limitations and Direction of Future Research

The findings in this study may be limited in their generalizability because, despite a broad recruitment strategy, only women from a high-socioeconomic-status population volunteered. Breastfeeding rates and duration within the sample were very high compared to the general U.K. population, and the women who took part were generally conscious of their food choices during pregnancy and how these may impact on the health and development of their baby. Further research is needed to establish generalizability of these results across samples.

Just over half of the participants (53.6%) were retained at the infant testing session; the attrition may be due to the fact that the study took place outside of the medical system, and without monetary or other exogenous incentives. Preliminary analyses found that the dropout and retained groups differed significantly with regard to infant head circumference and Apgar scores, although given the large number of participant variables collected, it is possible that these are chance findings. Indeed, while head circumference is significantly smaller for the dropout group (a potentially compromising feature), their Apgar scores are significantly larger (a positive feature), such that the two significant differences between groups are inconsistent.

Ideally, this study would have combined blood plasma levels of nutrients with the questionnaire data, however approaches such as ours based on food intake are an important complement to blood-sample studies, and provide a *direct* link between dietary intake and any outcome measures.

### Implications for Public Health

Physiological evidence suggests that most mothers do not have the necessary levels of DHA in their bodies during pregnancy (Hornstra, 2001). Appropriate supplementation has been demonstrated to raise both maternal and infant DHA levels (Helland et al., 2006) and breast milk DHA concentrations (Jensen et al., 2005). In this sample, one fourth of mothers were taking fish oil supplements during pregnancy and all of these participants were placed in the high-DHA intake group for analysis. As this group performed significantly better, this implies a case for supplementation to facilitate optimum cognitive development, particularly in mothers whose fish intake is low.

Current recommendations, specifically relating to DHA intake during pregnancy and lactation, suggest an average daily intake of at least 200 mg DHA (Koletzko et al., 2008) whether through increased oily fish consumption or via supplementation. Despite concerns around the levels of environmental toxins, such as mercury and dioxins, in oily fish, the majority of risk–benefit analyses conclude the benefits of increasing consumption of oily fish outweigh the risks (Cohen, Bellinger, Connor, & Shaywitz, 2005) and pregnant women should be advised as such.

Finally, this study has the unique advantage of directly linking maternal self-reported dietary intake of DHA to later cognitive outcomes. This justifies the implementation of practical dietary advice based on the study findings. From the specific findings of this study we suggest that pregnant women are advised on the most critical time points in pregnancy for ensuring sufficient DHA intake levels (for optimal infant cognitive development), which would appear to be during the third trimester.
